# Trust-based altruism facing new contexts: The Vyegwa-Gika pygmies from Burundi

**DOI:** 10.1371/journal.pone.0204321

**Published:** 2018-10-01

**Authors:** Cristina Acedo-Carmona, Enric Munar, Antoni Gomila

**Affiliations:** Human Evolution and Cognition Group (EvoCog), University of the Balearic Islands, IFISC, Associated Unit to CSIC, Cra. de Valldemossa, Palma de Mallorca, Balearic Islands, Spain; Universidad Loyola Andalucia, SPAIN

## Abstract

The community of Pygmies settled in Vyegwa-Gika provides an exceptional case study to test the role of trust in the evolution of altruism. The Vyegwa-Gika Pygmies were forced to migrate from rainforests to the savanna, changing quickly their environment, culture, and socio-economic situation. Despite the high level of poverty they suffer in this new settlement, we found evidence of strong altruistic attitudes toward trustees when playing an economic game. In addition, Vyegwa-Gika Pygmies keep small personal trust networks despite the fact they share frequent social interactions within the community. These results indicate the great effectiveness of personal trust in fostering altruism, even if the circumstances make it difficult to establish such kind of affective bonds. A theory of the evolution of altruism should therefore also account for the evolution of psychology of trust, as a key element in the process.

## Introduction

Altruism is a striking feature in humans compared to other species. Many of the explanations of its evolution focus on altruistic behaviors toward relatives–*biological* altruism [1, 2, 3]; toward group members–genetic [4] *and* cultural [5] *group selection*, *parochial* altruism [6]; or toward any other people by reciprocal cooperation–*reciprocal* altruism [7, 8], *altruistic* rewarding [9]–or indirect reciprocity–*altruistic* reputation [10, 11, 12], *altruistic* punishment [13, 14].

However, a proper explanation of the evolution of altruism should also consider the evolution of the psychological mechanisms that may generate such behaviors [15]. Thus, for example, it has been proposed that cooperation by reciprocity requires a psychological mechanism to estimate similar exchanges. This mechanism would give rise to the selection of reciprocating partners [16]. A different approach, the one we favor, focuses on personal trust, understood as the positive expectations about known people’s behavior, as a key psychological mechanism in this regard. Personal trust makes humans develop affective bonds beyond kin, and this feeling of affiliation toward trustees promotes to behave in favor of them, to expect them to do the same for us, and so to prefer them for reciprocal exchanges [17, 18]. Previous studies have also shown that personal trust can extend cooperation beyond trustees within a group, in an emotion-driven, non-strategic way, which leads to higher levels of group cooperation and cohesion [19, 18, 20, 21, 22]. For this reason, we have argued that personal trust is an evolutionarily acquired psychological trait, an adaptation to human sociality that played an important role in the evolution of cooperation [20, 21].

Evidence that a trait is an evolutionary adaptation may be found when it shows slow and light responsivity to fast environmental or cultural changes in the short term, while it delivers benefits in the long run, to become a universal trait. In fact, the universal character of personal trust was already suggested by the common patterns found in the personal trust networks across different cultures [20, 21, 22]. This approach finds further support in the fact that humans have lived in small groups most of their evolutionary history [23], and in the cognitive limitations to handle too much social information [24, 25].

In other words, if personal trust is an adaptation for human social life, it should be a robust mechanism, one that can be similarly found in all societies, and that plays its role regardless of education, wealth, or religiosity. Some cross-cultural evidence of the role of personal trust in fostering and channeling cooperation has already been provided [20, 22], while its role has also been observed to be more important in conditions of scarcity, where market exchanges are difficult or non-existent [20, 21]. However, this cross-cultural evidence refers to communities with a long history of shared living. In this study, we will consider whether personal trust keeps its role even when a community undergoes drastic environmental and cultural changes in a very short time. This is the case of the Pygmies of Viegwa-Gika from Burundi, who were forced to move from their rainforest traditional area to their current poor savanna setting. The situation of these Pygmies offers us an opportunity to test some predictions of our account. Firstly, even if a group undergoes important changes, the personal trust networks continue bonding its members and structuring their cooperative exchanges. Conversely, we expect cultural practices to change quickly when the circumstances change, while personal trust attitudes do not, even when such attitudes do not seem to be efficient enough to help thrive in a new environment in the short term. Thus, our goal was to study the role of personal trust psychology in these Pygmies’ new way of life.

Accordingly, given our view of personal trust as an evolutionary adaption to human social life, we hypothesize that: (1) Vyegwa-Gika Pygmies’ altruistic attitudes toward their personal trustees will be maintained despite their harsh living conditions; (2) but their personal trust networks will not change much, resembling those that existed in rainforests, instead of becoming bigger, as this would be more convenient in Vyegwa-Gika.

To test these hypotheses, we first asked Vyegwa-Gika Pygmies about their personal trust networks, and next, we invited them to play an economic game, either with somebody they trusted, or with somebody they do not, in order to find out whether trust still fostered altruistic cooperation in this new situation. As background, we explain first briefly below some important details about these groups and the economic, social and cultural changes they have undergone from their life in rainforests to their current settlement in the Vyegwa-Gika savanna. This information will help us later to contextualize the results of this study and be able to interpret them.

## Pygmies’ background and current situation

**Fig 1:**
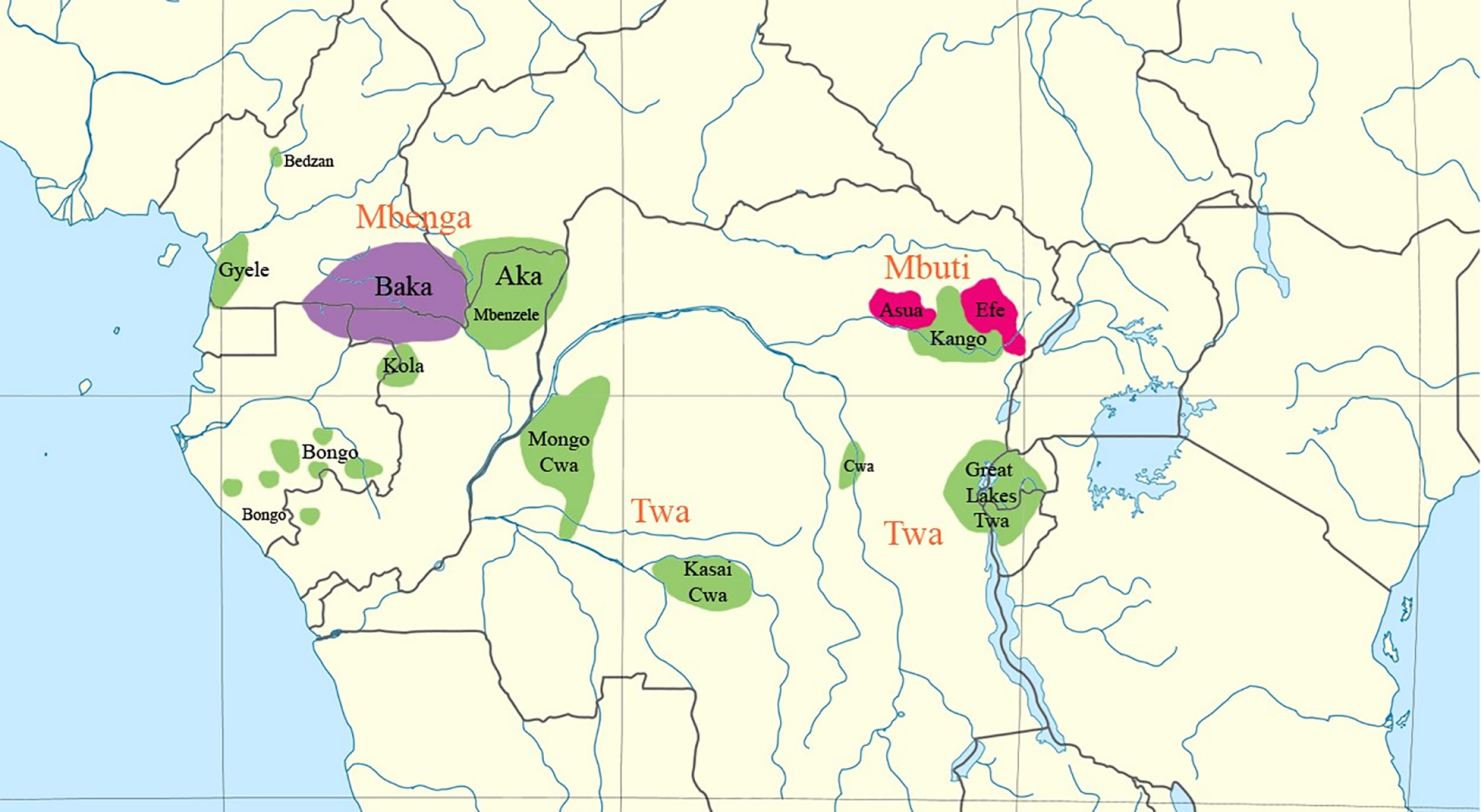
Distribution of Pygmies and their languages according to Bahuchet (2006). Green: Bantu, purple: Ubangian, red: Sudanic. This file is licensed under the Creative Commons Attribution-Share Alike 3.0 Unported license. https://commons.wikimedia.org/wiki/File:Pygmy_languages_(Bahuchet).png.

The African Pygmies are groups of hunter-gatherers who have inhabited the Central Africa rainforests for thousands of years as semi-nomadic groups [26, 27, 28]. In Burundi, the Great Lakes Pygmies are believed to be the oldest ethnic group settled in the country [29], although they currently represent just the 1% of the Burundi current population (approx. 80,000 Batwa). They are called “Batwa”, that is the plural of “Twa”, a term that refers to the Pygmies that inhabit the Great Lakes region (Fig 1). Pygmies that live in Burundi think that this denomination is used by other ethnic groups in a derogatory way, and they prefer to name themselves as "Abaterambere" (those who are developing themselves). Nevertheless, the term “Batwa”, far from having this connotation, will be used henceforth in the text to refer to the Pygmies from Burundi.

In general, the Pygmies in rainforests have been considered as one of the so-called "immediate-return" societies [30]. Unlike "delayed-return" societies of farmers, pastoralists and capitalists, which invest time to get a product, "immediate-return" societies of hunters and gatherers usually get an immediate return for their work, consume their products daily and do not accumulate stocks [31]. The Pygmies’ societies were also egalitarian [30]. They had not authority figures, as it generally happens in immediate-return societies [32]. Although some people could stand out for having some skills, they did not have a higher status than others because leadership was not an institutionalized figure. In rainforests, Pygmies lived in small groups [31]. The groups, settled in “camps”, were composed of about 10 families and between 50 and 60 people [31], although group sizes could vary temporarily: during hunting periods, for example, Pygmies had more chances of forming larger groups, but they usually separated for gathering. Often, individuals were bonded by clans, although people could change their clans. The social basis was first the nuclear family and then the camp. When sons got married, they formed their own families elsewhere [32, 33]. Small groups allowed them to create personal trust bonds strong enough to ensure mutual support in an environment where it was crucial to survive [34]^,^ and flexible enough to get adapted to a nomadic lifestyle [30].

**Fig 2:**
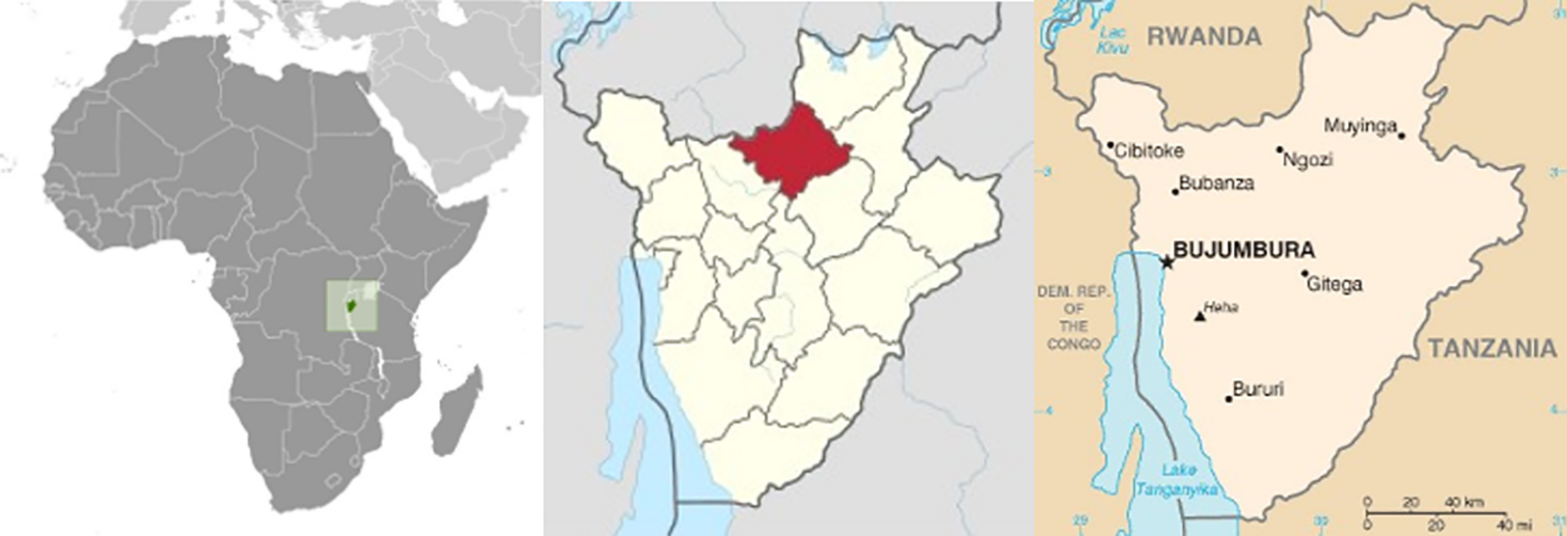
«Burundi location map» on the right, province of Ngozi in the center, and Ngozi city on the left. Images come from Wikipedia Commons available under license CC BY 3.0. https://commons.wikimedia.org/wiki/File:Burundi_location_map.svg#/media/File:Burundi_location_map.svg https://commons.wikimedia.org/wiki/File:Burundi_-_Ngozi.sv https://es.wikipedia.org/wiki/Burundi.

However, an excessive exploitation of new territories for agriculture and livestock by other ethnic groups who live in this territory (Bahutu and Batutsi [35, 36, 37]) caused the deforestation of the rainforests where Batwa lived. To be preserved, rainforests were declared natural parks or conservation areas during the 20th century, and Batwa were forced to look for other places where to live. As a result, their way of life was also stripped. In 1976 some of them moved to the savannah environment of Vyegwa-Gika, where the Burundian Government offered them farmlands. Vyegwa-Gika is located in the province of Ngozi, in the North of Burundi, about 6 kilometers from the city of Ngozi (Fig 2).

The Vyegwa-Gika settlement is now inhabited by about 150 families, around 900 people in total. Batwa in Vyegwa-Gika preserve the communal lifestyle from rainforest times, they meet often and share frequent common activities but now they interact within larger groups. Political parties have now been introduced in Vyegwa-Gika. Officials–the “Head of the hill” and “Councilor”–are elected for a five-year mandate in democratic elections where only those who are registered can participate. However, many Batwa in Vyegwa-Gika are not registered yet. Thus, Batwa are now in the process of accepting and respecting the authority of these elected officials. Nevertheless, this attitude of respect for authority is influenced by the fact that Batwa are poor people and *the head of the hill* has resources to help them if necessary. Unlike what happened in rainforests, in Vyegwa-Gika some Batwa have different levels of power to access to resources.

In addition to these social changes Batwa have also suffered a great change in terms of their independence about economic resources. In rainforests, they survived with the tools they built for hunting, they knew the places where animals used to move, ate antelopes and insects, and gathered honey, wild fruits, mushrooms, roots and plants. They recognized the properties of rainforest plants, to the point of becoming traditional healers for other ethnic groups. They also exchanged forest products for agricultural ones from other ethnic groups. In this way, they were respected by the other groups. In rainforests, they had all they needed but outside of this environment they became completely vulnerable. The current generations of Batwa in Vyegwa-Gika are beginning to adopt a sedentary lifestyle. Women are potters and start to cultivate, almost without resources, in common lands and in small spaces around their houses. Currently, women are also learning to breed livestock (goats, cows and rabbits). But the market for pottery products is very limited and they do not have enough resources to live from an economy based on agriculture and livestock. That's why men usually work as labor for other ethnic groups. Many of them still prefer to get an immediate profit for their services. Therefore, while Burundi is a poor country–around 80% of its inhabitants live under the poverty line [38] and they have a subsistence economy [39]–, poverty is higher for Batwa [38]. In addition, Batwa now barely relate to other ethnic groups living in nearby settlements, apart from working for them. The discrimination toward Batwa has increased: 90% of Batwa have problems to access to land, 89% of them do not have access to healthcare and 78% of them are illiterate [29]. They are described by Burundians as unfit to share a meal or drink with or get married to [29]. Consequently, Batwa have changed their social status from respected people when they lived in rainforests [40] to despised people in the new settlements. Thus, moving to the new settlement made these Pygmies plunge into poverty and marginalization and deprived them of its culture, created around the tropical forests. In the new environment, they have partially adopted habits, customs, beliefs, norms and values from other ethnic groups according to the new situation they live [33].

In summary, the Vyegwa-Gika Batwa have suffered abrupt economic, social and cultural changes in a short period of time.

## Empirical study

Once contextualized the situation that Vyegwa-Gika Batwa have faced, we proceed to detail the methods used in the empirical study that we conducted and the results obtained.

### Methods

#### Ethics statement

This study was approved by the Ethics Committee of the University of the Balearic Islands. We contacted the Rector of the Ngozi University and we asked for any concerns about doing research with Pigmies, and we were said that there were none.

Verbal informed consent was obtained from participants prior to participation, as approved by the Ethics Committee. Written consent was not obtained because most of the inhabitants of Vyegwa-Gika cannot read or write. Participants were asked by the students of the University of Ngozi if they wanted to participate in a research from the University of Spain that consisted of performing a task for which they would receive a prize. They were duly informed about what the investigation was and were said that their participation was totally voluntary and that they could stop participating whenever they liked. The people who were interested in participating gave their names and played the game in a building built in the settlement for community activities. Once the task began, participants’ personal data were collected in a form where were also registered their answers about the questions asked in the game.

The research was carried out by taking advantage of a customary visit of two NGOs, one from the Balearic Islands and one from Burundi, with a long track of cooperation projects in the region, much appreciated by the community (these NGOs provide tools, animals to breed, infrastructure works). The collaboration of these highly respected NGOs was essential for the Batwa to participate in the study.

#### Participants

A total of 58 Batwa settled in Vyegwa-Gika played an economic game. The only requirement for participants was to be able to do arithmetic operations. For this, we only recruited people who showed they at least knew how to add. They were assigned to one of the two different roles in the game: 30 of them played the role of *givers* and 28 of them played the role of *recipients*. The group of *givers* was composed by 13 males (43.3%) and 17 females (56.7%); and the group of *recipients* was composed by 11 males (39.29%) and 17 females (60.71%). About the economic situation of participants, 16.67% of *givers* declared being poor, 26.67% “more or less ok”, 53.33% “ok” and 3.33% “very well”; and 13.33% of *recipients* declared being poor, 40% “more or less ok”, 40% “ok” and 6.67% did give us any information on this issue. We must point out that these “economic levels” were based on their self-perceptions and quantified according to the words they used, but this information must be placed in the context of high poverty that practically all Batwa suffer in Vyegwa-Gika.

#### Procedure

About collecting trust networks:

We decided to elicit the participants’ trust networks as a good way to measure some features of trust relationships in this settlement [41, 42]. To identify the *givers’* personal trust networks, we tried to identify trustees using 3 hypothetical situations since the concept of trust is too abstract to ask about it to the participants directly. Thus, we asked participants to name a) *who they would leave the care of their goats* (in the case of women, because just women are cattle breeders) *or their beer boxes* (in the case of men, because beer is a very valuable product among men), *if needed*; b) *who they would lend their hoes* (in the case of women because farmers usually are women) *or their machetes* (in the case of men), *if needed*; c) *who they would leave the care of their huts or houses*, *if needed* (in the case of men and women). The answers to these questions provided a proxy for the participants’ personal trust networks. In addition, participants told us about the type of bond they kept with the mentioned people (trustees) and the daily time they spent together. The study was carried out by students of the University of Ngozi in the participants’ native language (Kirundi). Researchers trained these students to perform the research properly and were given a form of instructions where to also register the responses. These forms can be seen in Supporting Information (S1 Tables).

About playing the economic game:

In the game, we offered to *givers* four units of something very appreciated by them without telling what it was until all participants completed the game. The problem that we solved with the unknown reward was the possible heterogeneity in Batwa’s preferences. *Givers* had to donate three out of these four units to a *recipient* and keep one for them. At this point, *givers* had to decide between giving these three units to someone of their previously mentioned trustees (without knowing who in particular), or to another inhabitant of the settlement (without knowing who in particular either). *Givers* were told that *receivers* had to give back at least one the units and that if the *receiver* was one of their trustees, they would receive just the units that the *recipient* decided to give back to them. However, if *givers* gave the three units to any other person from the settlement, they would receive twice as much as the *recipient* decided to give back to them (therefore, two units as a minimum). In other words, the exchange was set in such a way that *givers* could gain more in return by giving to non-trustees than to trustees. In addition, *givers* were asked to say the number of units they expected from *recipients*.

**Fig 3:**
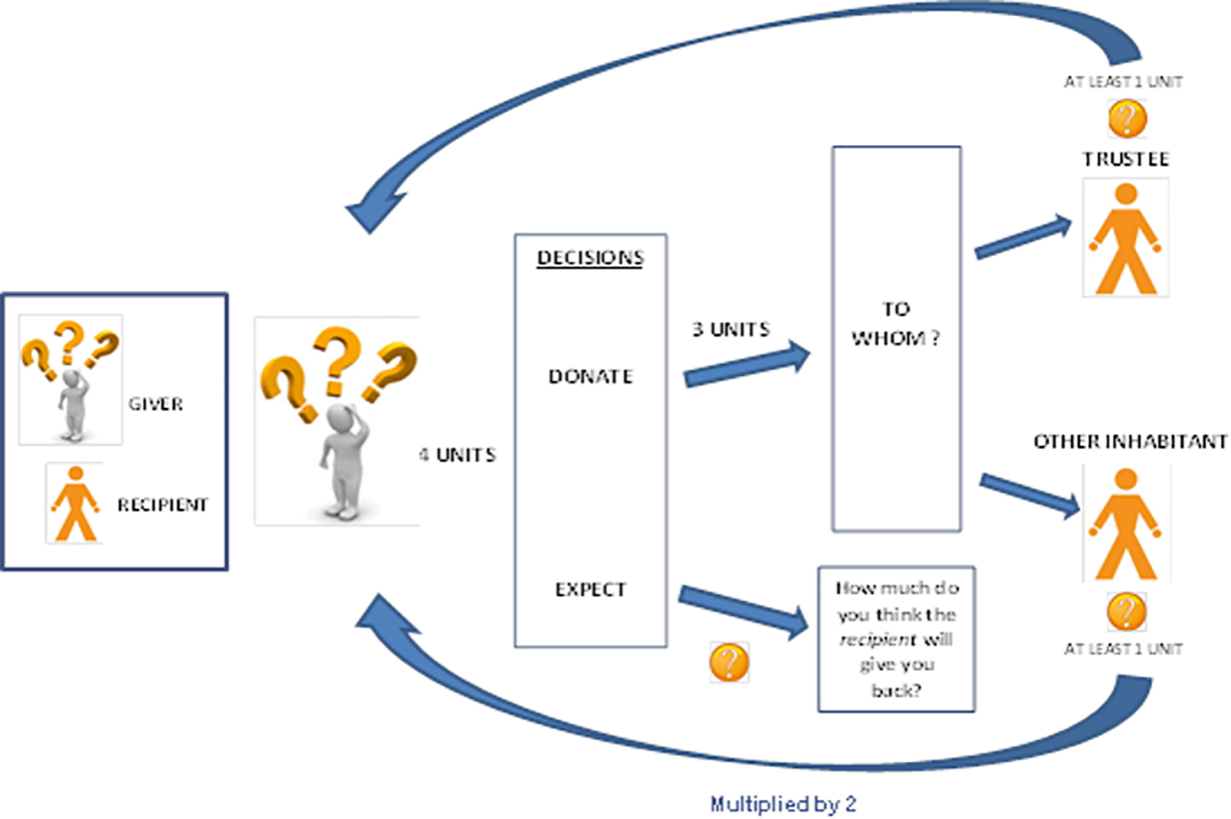
Representation of the game.

Once all *givers* made their decisions, *recipients* decided how many units to give back to *givers*, knowing whether the *giver* was or not a person close to them (closeness depended on whether *recipients* were or not *givers’* trust networks respectively). Both *givers* and *recipients* were informed that they would never know the identity of their game partners. The units received were changed for food at the end of the game. The game is represented in Fig 3 and in S1 Tables appear the forms used to explain the game and obtain data.

The research was carried out in Vyegwa-Gika taking advantage of the visit of members of two NGOs: "*Veïns sense fronteres*", from the Balearic Islands (Spain), and "*ABI (Abazimyamuriro Bazira Imbibe)*", from Burundi. Both NGOs have been working in this community for years, providing valuable materials for Pygmies, such as different tools, building materials to build their houses, animals for breeding, and seeds for cultivation. The Batwa associate these NGOs with such valuable elements for their community. For this reason, the participants assumed that the prizes would be some of the goods these NGOs provide them with. Therefore, the game incentive, the prize, was very real for them.

To make sure that participants could understand the game properly, we allowed playing only those able to count and add. In addition, the possible outcomes were explained with the use of stones. Each stone constituted a unit of the prize that later they would receive. Once explained this, they were asked several questions about what they would receive according to the possible responses of the *recipients* in both conditions, to make sure they understood the game properly. The explanations and the answers were repeated as many times as necessary until it was proven that they understood the game.

### Results

#### Trust networks

**Fig 4:**
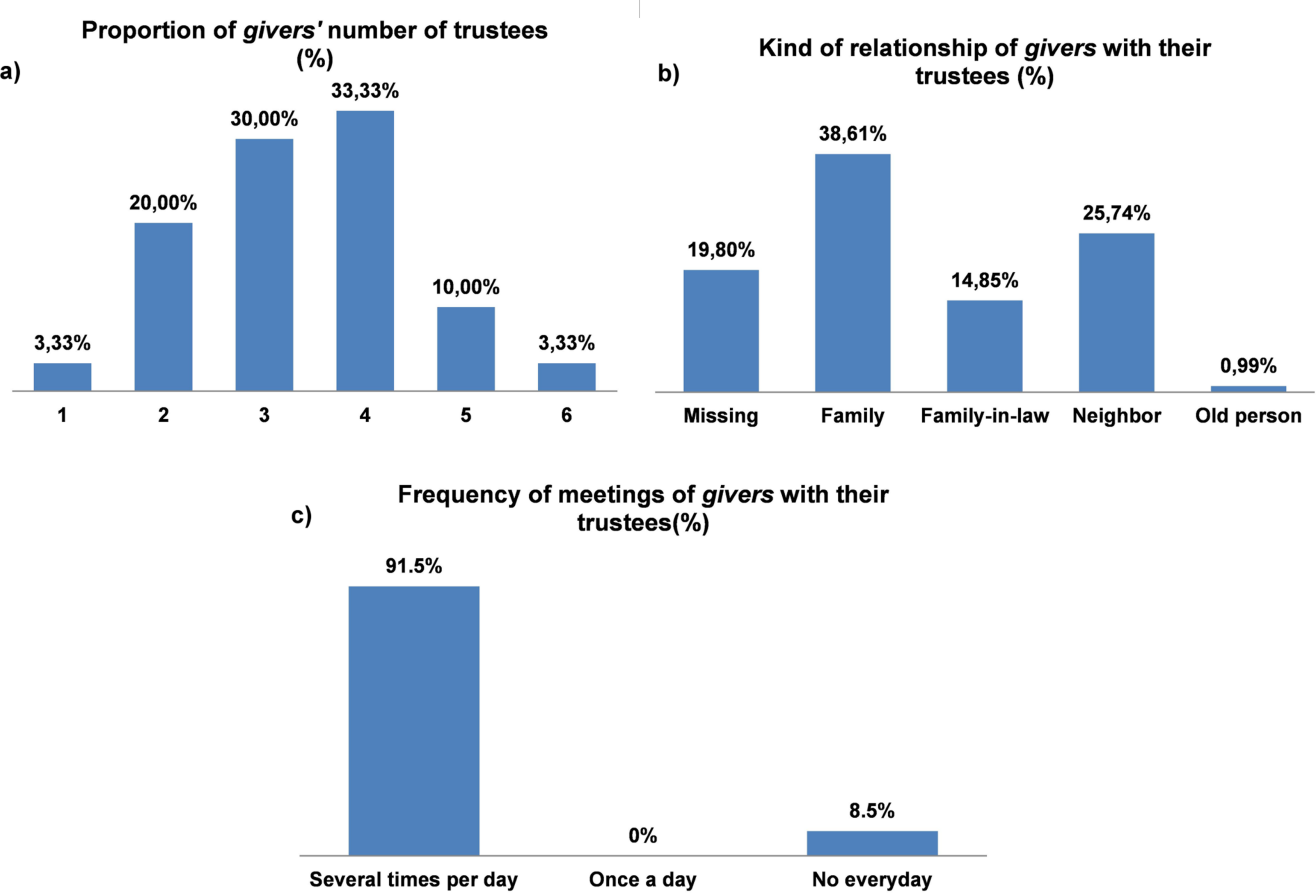
a) Proportion of *givers* about the number of trustees mentioned; b) Kind of relationship of *givers* with their trustees; c) Frequency of meetings of *givers* with their trustees.

The results from personal trust networks showed that the mean of trustees by *giver* according to the three previously posed situations was not very high (Mean ± SE = 3.37 ± 0.206, N = 30). The most frequent numbers of trustees among the *givers* were of 3 and 4 (30% and 33.33% of all the cases respectively) (Fig 4A). The 91.5% of trustees met *givers* several times a day (Fig 4C). These low numbers of *givers*’ trustees contrast with the frequency of the daily activities that Vyegwa-Gika Batwa share, their coexistence within larger groups and the frequency of their social interactions: women meet to work in pottery, singing and dancing, several times a month to produce ceramics for their own use and selling to tourists; women also meet and sing when they cultivate communal lands; men go out in the evening and drink beer together; the Vyegwa-Gika Batwa men and women have also been working together to build new houses thanks to the aid of NGOs; and they have also built a common house to meet and discuss community affairs.

These small network sizes, though, resemble those previously described of rainforest Batwa, mostly limited to the members of the nuclear family or close camp members [30, 33]. In fact, 51.46% of trustees were relatives (genetic relatives: 38.61%; relatives-in-law: 14.85%) and 25.74% of them neighbors (Fig 4B). It is as if trust continues to be established among the same kind of social relationships, despite the changes taken place in Vyegwa-Gika, where marriage breakdowns have increased and individuals interact frequently with a larger group of non-relatives.

**Fig 5:**
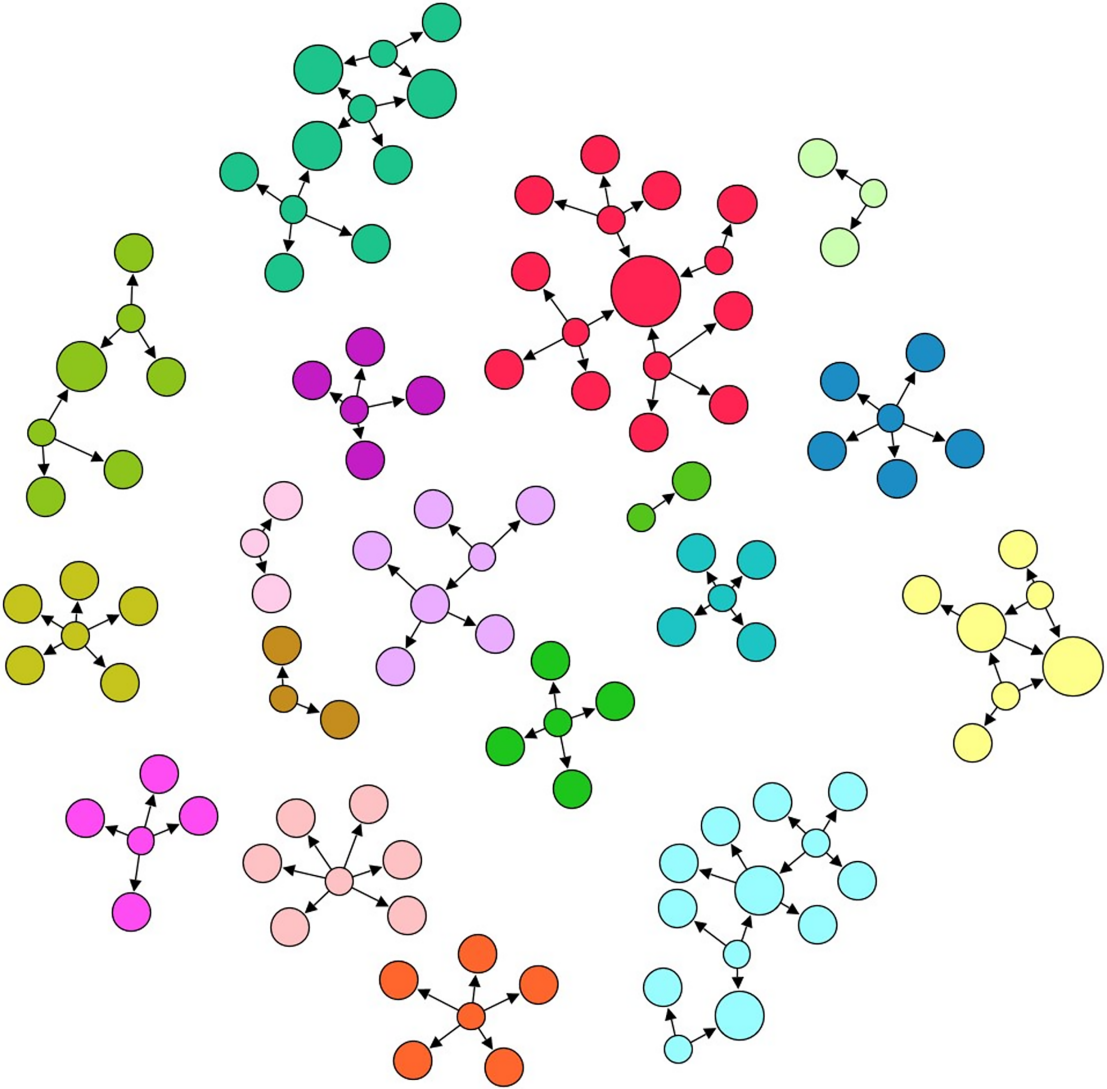
Representation of the *givers’* personal trust networks. The node size refers to the in-degree level (that means the number of trusters of each node). Arrows go from *givers* nodes to their trustees.

In addition, personal trust networks were poorly connected to each other. Few individuals were mentioned repeatedly in several personal trust networks. In fact, nobody was trusted by most participants. Thus, our results of network analysis with Gephi software [43] indicate that the largest trust network had not more than 15 members. Likewise, the highest *betweenness* coefficient [44] was 6 –betweenness coefficient measures how often a node appears on the shortest paths between nodes in the network. In other words, the trustee who is the greater connector for all the pairs of trust network members was a connector just on 6 occasions. This low coefficient indicates that the participants’ personal trust networks are small and disconnected. What is more, the individual who did stand out in this sense had not a very remarkable position, as gauged by several other network measures: an *in-degree* coefficient [45] of 4 nodes–in-degree coefficient measures how many people trust this individual; a *level of* authority [46] of 0.026 (range 0–1)–level of authority measures how valuable is the information stored in that node, referred in this case to the level of trustworthiness of his trustees; and an *eigenvector* centrality [47, 48] of 0.198 (range 0–1)–eigenvector centrality measures the importance of a node in the network based on their connections. The lack of people who stand out for being trustees for many other group members is not consistent either with the authority figures found in Vyegwa-Gika (the Head of the hill or the Councilor), but it is consistent with the absence of an authority figure, as happened among rainforest Batwa [30]. In the Fig 5 participants’ personal trust networks are represented.

Finally, same gender matters for personal trust: most of the *givers’* gender coincided with that of their trustees (Pearson’s chi-square, χ^2^ = 11.01, p < 0.005): 82.35% among females and 76.92% among males.

#### Economic game

The economic game results showed that, despite the harsh life conditions of Batwa in Burundi, particularly in the area of Vyegwa-Gika, 93.33% of the *givers* decided to donate their 3 units to a trustee, risking getting back just 1 unit instead of the 2 units they would get from a non-direct trustee, as the minimum return. In other words, *givers* made an altruistic decision, one that benefited the trusted recipient, to a cost for themselves.

**Fig 6:**
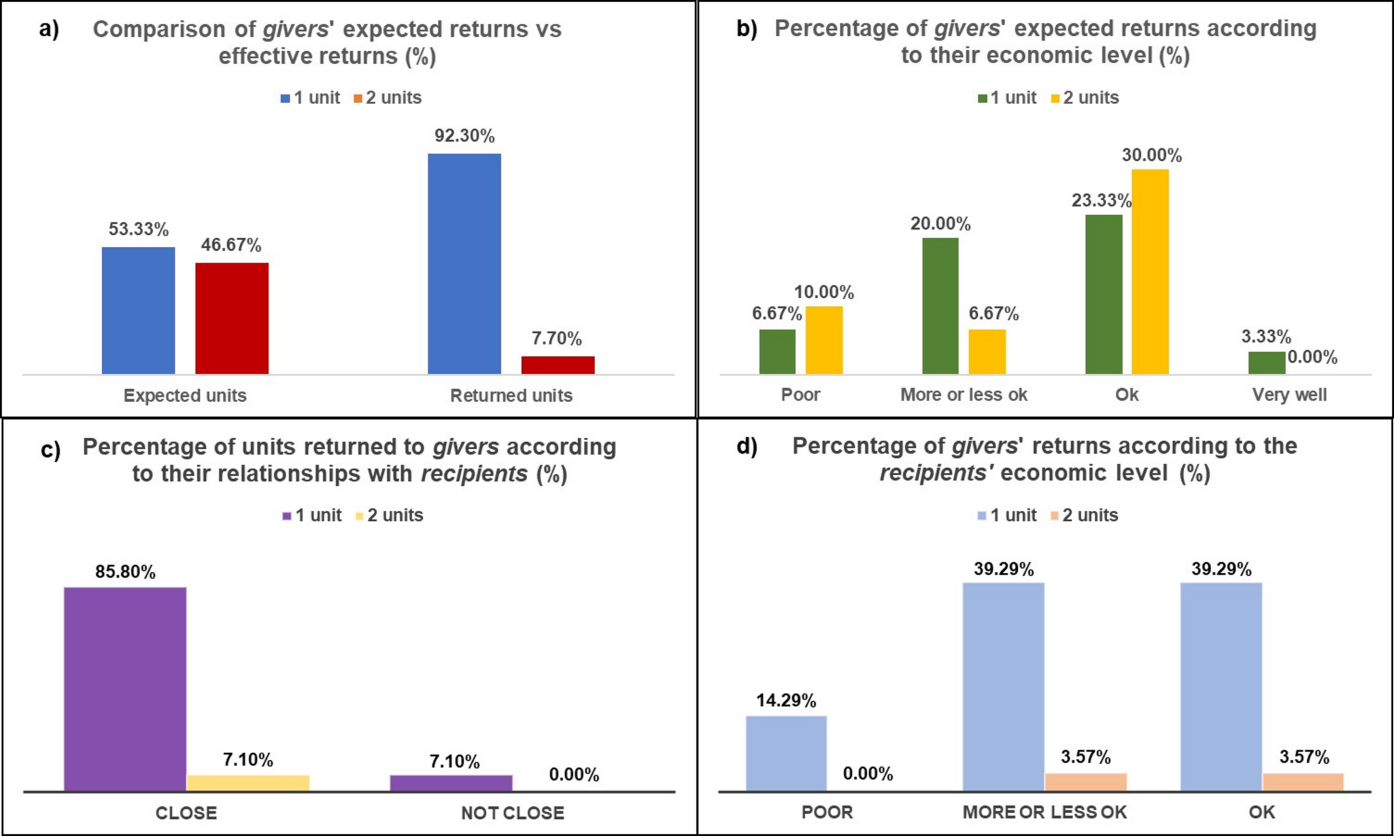
a) Proportion of *givers*’ expected returns versus effective returns from *recipients*; b) Proportion of *givers*’ expected returns according to their economic level; c) Proportion of units returned to givers according to their relationships with *recipients*; d) Proportion of *givers’* returns according to the *recipients’* economic level.

The altruistic character of decisions is made clearer upon consideration of the *givers*’ expectations about the number of units to be returned by *recipients* (we remember that most of the *recipients* were trustees: 93.33%): 53.33% of *givers* expected the minimum (1 unit) from *recipients* and 46.67% of them expected 2 units. In the first case, because *givers* decided to donate their units assuming that they might receive fewer units back; and in the second case, because their expectation was that the *recipients* would behave altruistically, even knowing the severe poverty they suffer. Remarkably, we did not find significant differences–Pearson’s chi-square–between *givers’* expectations and the real *recipients’* behavior, (χ^2^ = 0.01, p < 0.9), even if in the second case the expectation turned out to be an overestimation, given that 92.3% of *recipients* gave back 1 unit (Fig 6A). The expectations of *givers* about the number of units returned by *recipients* were neither related to the perception of their own “economic situation” because we did not find significant differences–Pearson’s chi-square–between these variables (χ^2^ = 3.33, p < 0.3) (Fig 6B). These results, then, suggests that *givers’* decisions were not strategic but rather an expression of a preference for their personal trustees. On the other hand, the dominant decision of *receivers* to return only 1 unit could be expected in people with such high level of poverty. In this case we did not find a relation between the number of returned units and closeness to the *giver* (Pearson’s chi-square, χ^2^ = 0.17, p < 0.7) (Fig 6C) nor between the number of returned units and *recipients’* perceptions about their own “economic situation” (Pearson’s chi-square, χ^2^ = 0.36, p < 0.9) (Fig 6D). The fact we did not find a relation between *givers’* and *recipients’* perceptions about their own economic situation and their game decisions does not surprise us because the level of poverty is very high for all Vyegwa-Gika Batwa. So, the different expressions they used to answer this question do not seem to respond to real differences in their economies.

## Discussion and conclusions

In summary, while Vyegwa-Gika Batwa have undergone many social and economic changes since moving to his new settlement, where they live in larger groups, personal trust remains a strong component of close-knit small scale interpersonal dynamics and relationships [49, 50]. In particular, the interaction of Batwa within larger groups, sharing time and many activities together, and having so frequent interactions, has not promoted larger, and more cohesive, personal trust networks [16], a result that is congruent with other studies [20, 51, 52, 53, 54]. By contrast, personal trust networks, so important to promote commitments of reciprocity and essential to survive in hard conditions of life, are limited to a small number of people (a mean of 3.37 people). This is a similar to that found in the small groups of rainforests Pygmies (according to hypothesis 2). In rainforests, small networks were adaptive, given the economic and environmental conditions of groups, which could become bigger for hunting periods. However, rainforest Batwa mobility and the flexibility in the configuration of their groups [49, 50, 52, 55] suggests that strong levels of trust and altruism happened only within small groups.

In the new habitat, on the other hand, small personal trust networks reduce the possibilities of getting support and cooperation in such a situation of harsher survival, although they generate strong ties. These ties increase altruistic decisions (not obligations), as evidenced by the game played by our participants, benefiting trustees at the expense of themselves even if the game was played anonymously and despite the *givers’* poverty (according to hypothesis 1). The level of poverty of the *givers* increases the cost of failing to obtain a benefit [56] when they decide to deliver part of the obtained units to a trustee instead of to another person of the settlement. Thus, this behavior indicates a high level of altruism among the members of trust networks. But this altruistic pattern toward *givers’* trustees is not reproduced by *recipients’* close people. Thus, trust induces altruism in a way that closeness does not, maybe because for *givers* it was possible to imagine the *recipient*, but not the other way around.

The absence in personal trust networks of salient trusted people for many others is also closer to rainforest social patterns than to the new situation where authority figures are being introduced by other ethnic groups. So, the difficulty to tolerate differences of authority within the community, characteristic of the egalitarian societies of rainforest Pygmies, remains in the trust psychology of Vyegwa-Gika Batwa. Accordingly, personal trust networks appear disconnected and without centralized nodes.

Thus, although Vyegwa-Gika Batwa have adopted cultural practices (festivals, ceremonies, rituals…), economic activities (agriculture and livestock) and social organizations (political parties, community associations …) from other ethnic groups, personal trust psychology seems to have maintained the old patterns acquired in rainforests, as expected if trust is an evolved adaption, universal trait, resistant to changes.

Finally, it is also striking the overrated positive expectations of many *givers* toward their trustees’ behavior. Despite knowing the situation of poverty of Vyegwa-Gika inhabitants, nearly half of the donors (46.67%) expected to receive back 2 from the 3 donated units. These overrated positive expectations from trustees, which reinforce trust and cooperation bonds among them, reveals an expectation of reciprocal generosity that was essential to survive in rainforests.

Considering these results, our study supports the view that personal trust is an evolutionary adaptation to social life. Although this psychological mechanism may seem negative for survival in the short term from a strategic, self-interested point of view, it offers long-term advantages because it strengthens emotional ties of reciprocal cooperation even in adverse situations. While personal trust networks can adopt different shapes depending on environmental resources available [16], its evolutionary nature makes it robust and difficult to change, in contrast with the pace and dynamics of cultural change. Certainly, this approach requires further support and a cross-cultural approach may offer the best evidence to provide it.

## Supporting information

S1 Tables Forms for the *givers* and *recipients* (in the French language). (DOCX)
