# Supplementary material for: Trust-based altruism facing new contexts: The Vyegwa-Gika pygmies from Burundi
Source: PLoS One. 2018 Oct 1;13(10):e0204321. doi: 10.1371/journal.pone.0204321 (PMC6166921; doi:10.1371/journal.pone.0204321)
Supplement: S1 Tables — (DOCX) [file pone.0204321.s001.docx]

**Supporting Information**

S1 Tables. Forms for the *givers* and *recipients* (in the French language)*.*

Forms for the *givers*

Form for the *recipients*
